# Emission Quenching in Tetraphenylfuran Crystal: Why This Propeller-Shaped Molecule Does Not Emit in the Condensed Phase

**DOI:** 10.3390/molecules27020522

**Published:** 2022-01-14

**Authors:** Ljiljana Stojanović, Rachel Crespo-Otero

**Affiliations:** 1 School of Physical and Chemical Sciences, Queen Mary University of London, Mile End Road, London E1 4NS, UK; l.stojanovic@qmul.ac.uk; 2 Department of Physics and Astronomy, University College London, Gower Street, London WC1E 6BT, UK

**Keywords:** aggregation-caused quenching, aggregation-induced emission, solid-state luminescence, propeller-shaped molecules, excited states

## Abstract

Due to their substantial fluorescence quantum yields in the crystalline phase, propeller-shaped molecules have recently gained significant attention as potential emissive materials for optoelectronic applications. For the family of cyclopentadiene derivatives, light-emission is highly dependent on the nature of heteroatomic substitutions. In this paper, we investigate excited state relaxation pathways in the tetraphenyl-furan molecule (**TPF**), which in contrast with other molecules in the family, shows emission quenching in the solid-state. For the singlet manifold, our calculations show nonradiative pathways associated with C-O elongation are blocked in both vacuum and the solid state. A fraction of the population can be transferred to the triplet manifold and, subsequently, to the ground state in both phases. This process is expected to be relatively slow due to the small spin-orbit couplings between the relevant singlet-triplet states. Emission quenching in crystalline **TPF** seems to be in line with more efficient exciton hopping rates. Our simulations help clarify the role of conical intersections, population of the triplet states and crystalline structure in the emissive response of propeller-shaped molecules.

## 1. Introduction

The optimisation of highly emissive organic molecules has become a milestone in the technology of optoelectronic materials. Due to the presence of defects and the stabilisation of specific intermolecular interactions, such as π-π stacking and hydrogen bonds, emission quenching is very common in the condensed phase. In the last decade, several organic crystals showing a significant enhancement of luminescence have been reported. The term aggregation-induced emission (AIE) has been commonly used to describe this phenomenon. The term solid-state luminescence enhancement (SSLE), proposed by Gierschner et al. better highlights the synergistic effect of inter and intramolecular interactions on emission in the solid-state [1].

Two complementary models are widely used to explain AIE and SSLE: restricted access to the conical intersection (RACI) and restriction of intramolecular motions (RIM). According to the RACI model, the conical intersections, which act as funnels for internal conversion (IC) to the ground state, are destabilised in the crystal environment due to the steric hindrance, decreasing the internal conversion rate, and consequently, increasing the fluorescence yield [2,3,4,5,6]. This model is appropriate when a molecule possesses enough energy to explore regions of excited-state surfaces with strong nonadiabatic couplings between the ground and excited states. However, when the energy barrier prevents relaxation through IC, a part of the population can be transferred through vibrational transitions to the ground state, as a result of overlaps between ground and excited state vibrational wave functions. Shuai et al. have derived a formalism based on Fermi’s golden rule, which proves that low-frequency vibrations enhance the IC rate [7]. In crystal environments, where low-frequency motions are partially hindered, the internal conversion rates decrease. This is the basis for the RIM model.

**Figure 1:**
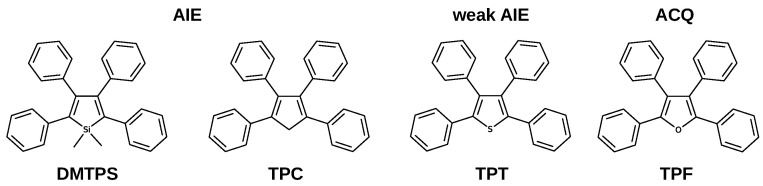
Structures of propeller-shaped molecules with different emissive response.

Propeller-shaped molecules are typical systems with an enhanced emission response in the condensed phase [8]. These chromophores are composed of a static core, typically a five-membered aromatic ring, surrounded by phenyl rings as rotors. Several propeller-shaped systems (Figure 1) exhibit significant fluorescence yields in the crystalline phase [8,9,10].

It is expected that propeller-shaped systems derived from furan and thiophene also show AIE properties, however, tetraphenylthiophene (**TPT**) is a weak AIE-gen, whereas tetraphenylfuran (**TPF**) exhibits aggregation caused quenching. We have recently studied the relaxation mechanism in crystalline **TPT**, concluding that active intersystem crossing pathways decrease emission efficiency, even though IC is hampered [10]. Our calculations show that the nature of the central atom can modify the nature of conical intersections involved in the main nonradiative pathways; when the central atom is modified from C, to S and O, the main nonradiative pathways change from puckering to bond breaking. **TPF** is the only member of the family with significant emission efficiency in solution (0.40). Upon crystallisation to nanoaggregates, the fluorescence is completely lost. According to experimental results, quenching is due to both an increase in nonradiative decay and to a smaller extent to a decrease of radiative decay upon aggregation [11].

Several modifications of **TPF** have been attempted to improve its radiative response. Contrary to **TPT**, **TPF** is not piezoemissive, even though the steric volume significantly decreases within a certain range of applied external pressure [12]. The introduction of bulky substituents in positions 3 and 4 of furan does not improve emissive properties either. However, oxidative ring-opening of furan produces 1,4-enedione [13], whose crystal has a significant fluorescence yield. Another successful approach is the design of 1,2,3,4-tetraphenyloxazolium (**TPO-P**) and 2,3,5-triphenyloxazolium (**TriPO-PN**) crystals derived from **TPF**, which have significant anion-$\pi^{+}$ interactions that suppress π-π stacking and minimise the intermolecular nonradiative pathways [14].

In this paper, we investigate the main excited-state radiative and nonradiative decay mechanisms of **TPF** in the vacuum, solution and crystal. We consider the effect of intermolecular interactions, vibrations and exciton formation, and compute reorganisation energies in three phases. By analysing the potential energy surfaces of the ground and excited singlet and triplet states, we identify the minimum energy intramolecular nonradiative pathways in vacuum and crystal. Our calculations show that the minimum energy nonradiative pathway is associated with the C-O bond elongation, however, a high barrier corresponding to the $\pi \pi^{*}$/$\pi \sigma^{*}$ intersection, prevents the $\pi \sigma^{*}$ state population and access to the conical intersection in both phases. In contrast with related systems, the intermolecular processes seem to play an important role in excited state relaxation in the **TPF** crystal.

## 2. Computational Details

To explain the light-activated processes in **TPF**, we considered its excited states in the gas phase, tetrahydrofuran (THF) solvent, and the crystal phase. The ground state (FC) and S${}_{1}$ minima were optimised with (TD-)DFT and ωB97X-D/6-31G(d) [15,16,17,18,19,20]. Several electronic structure methods were then assessed for the prediction of the absorption and emission energies. For the simulation of the dielectric environment of tetrahydrofuran (THF) (ϵ = 7.6), the polarisable continuum model (PCM) was used with the (TD)-B3LYP/6-31G(d) and (TD-)ωB97X-D/6-31G(d) methods, with the linear-response equilibrium variant for the excited states. Optimisations and single-point computations with DFT and TD-DFT were performed with Gaussian 16 [21].

We also considered wave-function methods; resolution-of-the-identity coupled-cluster with approximate second-order excitations (RI-CC2/aug-cc-pVDZ) [22,23,24,25], and complete active space perturbation theory (CASPT2) method [26,27,28]. The CASPT2 calculations were performed in the space of the configuration state functions obtained with SA-3-CASSCF(10,10)/6-31G(d) [29]. The active space was composed of 8 π orbitals with significant occupations and a bonding/antibonding pair of sigma C-O orbitals (Figure S1 in the Supporting Information). The CASPT2 computations were performed with 0.1 au imaginary shift and without an IPEA shift. The S${}_{1}$–S${}_{0}$ minimum energy conical intersections (MECIs) were optimised with the SA-2-CASSCF(10,10)/6-31G(d) level of theory, using the branching plane update method [30] implemented in the Molcas code [31]. The RI-CC2 computations were performed with the Turbomole v7.0 code [32].

The experimental crystal structure of **TPF** was retrieved from the Cambridge Crystallographic Database (the CCDC code is 1494293) and refined with DFT-periodic boundary conditions as implemented in Quantum Espresso [33]. The PBE-D2 functional was used with a plane-wave cutoff of 30 Ry and a Monkhorst-Pack k-point grid of (1 × 2 × 1), chosen according to the dimensions of the unit cell.

Clusters of 44 molecules were extracted from the optimised supercells for the subsequent QM:MM calculations with electrostatic embedding. The central molecule in the cluster was treated using the QM framework, whereas the surrounding molecules were modelled with MM. The QM region was relaxed whilst the MM region was kept fixed at its optimised lattice positions. FC and S${}_{1}$ geometries were optimised applying the ONIOM(QM:MM) method [34,35] using the Gaussian 16 software [21]. The QM region was treated using the ωB97X-D/6-31G(d) level of theory under the (TD-)DFT framework. The MM region was simulated with the Amber force field [36] using ESP charges derived from a vacuum HF/3-21G* calculation of the monomer. We also analysed the slip-stacked dimer with the shortest centroid using the ONIOM embedded cluster method (OEC) implemented in fromage [37,38]. The QM (selected dimer) and QM’ (environment) regions were simulated with TD-B3LYP/6-31G(d) and the second order (SCC-)DFTB [39] method, employing the mio-1-1 set of Slater-Koster parameters parametrised for the tight-binding SCC-DFTB Hamiltonian [40]. For the point charges we used the RESP charges obtained at ωB97X-D/6-31G(d) and PBE/6-31G(d) levels of theory, respectively. The DFTB calculations were performed with the DFTB+ program [39].

The S${}_{1}$–S${}_{0}$ MECI and T${}_{1}$–S${}_{0}$ crossing in the solid state were optimised using QM/MM with the interface between the Molcas and Tinker (version 6.3.3) codes. The QM region was described at the SA-2-CASSCF(10,10)/6-31G(d) level of theory, whereas the surrounding molecules were treated using the Amber force field. The pathways connecting FC, S${}_{1}$ and the crossing geometries in vacuum and crystal were created by restricted SA-2-CASSCF(10,10)/6-31G(d) optimisations of the S${}_{1}$ state by increasing the C-O bond length. Single point calculations with MS-3-CASPT2/SA-3-CASSCF(10,10)/6-31G(d) were performed on the optimised geometries. We obtained the diabatic representations by analysing the composition of adiabatic states in terms of excitations between CASSCF orbitals and connecting the states of the same type.

The spin-orbit coupling (SOCs) between the first three singlet and triplet states (S${}_{0}$–S${}_{2}$ and T${}_{1}$–T${}_{3}$) were computed at relevant geometries with the Molcas code, employing SA-3-(10,10)CASSCF/6-31G(d) ground and excited-state wave functions [31]. The SOCs were calculated using the components of matrix elements between singlets and triplets with quantum numbers $m_{l}\in \left\{-1,0,1\right\}$ as $|\langle S_{i}|H_{SO}|T_{j}\rangle |=\sqrt{\sum_{m_{l}=-1,0,1}|\langle S_{i}|H_{SO}|T_{m_{l},j}{\rangle |}^{2}}$.

The fluorescence rates ($k_{r}$) were evaluated using the Einstein equation for spontaneous decay from a state with emission energy (ΔE) and oscillator strength (*f*)

(1) $$k_{r}^{Ein}=\frac{2\Delta E^{2}f}{c^{3}}$$

where all variables and constants are represented in atomic units.

The intersystem crossing (ISC) rates ($k_{ISC}$) between S${}_{1}$ and T${}_{1}$/T${}_{2}$ states were evaluated based on the Marcus-Levich-Jortner model as [41,42,43,44]

(2) $$\begin{array}{c} k_{ISC}^{MLJ}=\frac{2\pi }{\hbar }|\langle S_{i}|H_{SO}|T_{j}\rangle {|}^{2}\frac{1}{\sqrt{4\pi \lambda k_{B}T}}\\ \sum_{n}exp\left(-s_{k}\right)\frac{s_{k}^{n}}{n!}exp\left[-\frac{{\left(\Delta E_{ST}+n\hbar \omega_{k}+\lambda \right)}^{2}}{4\lambda k_{B}T}\right] \end{array}$$

$\Delta E_{ST}$ is the energy gap between *T*${}_{m}$ and *S*${}_{n}$ states at their minima, λ is the total reorganisation energy of low-frequency normal modes ($\omega_{j}\leq 600\;{\mathrm{cm}}^{-1}$), *n* is the vibrational quantum number. The higher frequency modes ($\omega_{j}>600\;{\mathrm{cm}}^{-1}$) are represented by a single effective mode with a frequency $\omega_{k}$ obtained as

(3) $$\omega_{k}=\frac{\sum_{j}\omega_{j}s_{j}}{\sum_{j}s_{j}}$$

where $s_{j}$ and $\lambda_{j}$ are the Huang-Rhys factors and reorganisation energies of these high frequency modes. The Huang-Rhys factor for the effective mode ($s_{k}$) is calculated as $s_{k}=\sum_{j}\lambda_{j}/\hbar \omega_{k}$. These values were calculated based on the normal modes of the monomers for the S${}_{1}$ and T${}_{2}$ minima at the TD-B3LYP/6-31G(d) level of theory in the gas phase, solution, and solid state using the Dushin code [45].

Exciton couplings (*J*) were computed applying the Troisi’s diabatisation scheme based on the transition dipole moments of isolated molecules and dimers as implemented in fromage [38,46]. This method takes into account short-range (exchange, orbital overlap, charge-transfer) and long-range (Coulomb) interactions. The exciton hopping rates ($\nu_{ij}$) between monomers *i* and *j* can be estimated based on the Marcus model [41] as

(4) $$\nu_{ij}=\frac{J_{ij}^{2}}{\hbar }\sqrt{\frac{\pi }{\lambda k_{B}T}}exp\left[-\frac{\lambda }{4k_{B}T}\right].$$

$J_{ij}$ is the exciton coupling, λ is the reorganisation energy for exciton hopping between monomers, *ℏ* is reduced Planck’s constant, $k_{B}$ is Boltzmann’s constant, and *T* is the temperature. The reorganisation energies are computed as sum of reorganisation energies within ground and excited states ($\lambda =\lambda_{g}+\lambda_{ex}$), obtained at the TD-ωB97X-D/6-31G(d) level of theory on monomers of **TPF**.

## 3. Results and Discussion

### 3.1. Vertical Excitations and Radiative Mechanisms

The experimental absorption spectrum of **TPF** in THF solution features an intense band at 327 nm and a low-intensity band at 270 nm, whereas fluorescence peaked at 383 nm [11]. The experiments do not show a shift in the emission energy due to crystallisation. We tested the performance of single-reference (TD-DFT and RI-CC2) and multi-reference methods (CASPT2/CASSCF) for the description of **TPF** absorption (Franck-Condon point, FC) and emission spectra ($S_{1}$ minimum) in vacuum, solution and crystal phase (Table 1). The CASPT2 and CC2 excited states were computed using the geometries optimised with (TD-)ωB97X-D/6-31G(d), whereas TD-DFT (B3LYP and ωB97X-D) excitation energies are computed at their respective $S_{0}$ and $S_{1}$ minima.

The absorption band peaked at 327 nm originates from the excitation to the bright 1${}^{1}\pi \pi^{*}$ state. Both single-reference and multi-reference methods predict reasonably well the excitation and emission energies. According to the TD-DFT results, the absorption and emission shift negligibly going from vacuum to crystal. This is in line with the experiments that showed that the emission energies do not changed upon crystallisation.

According to the experimental results, the decrease in the fluorescence quantum yield going from solution ($\Phi_{f}$ = 0.40) to the aggregate phase ($\Phi_{f}$ = 0.0) originates simultaneously in a large increase of nonradiative (from $8.8\times 10^{8}$ s${}^{-1}$ to $166\times 10^{8}$ s${}^{-1}$) and a decrease of radiative rate (from $5.88\times 10^{8}$ s${}^{-1}$ to $0.67\times 10^{8}$ s${}^{-1}$) (Table 2). The estimated radiative emission rate using the Einstein equation (Equation (1)) is in very good agreement with the experimental value in the solution of THF. The predicted emission rate is only slightly lower in the solid state in comparison with the value in solution. This is in contrast with the significant decrease observed experimentally, which our model was not able to capture.

### 3.2. Nonradiative Relaxation Mechanisms

**Figure 2:**
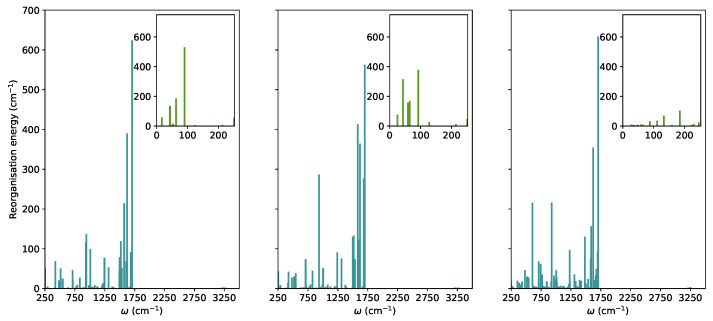
The reorganization energies for the relaxation from the S${}_{1}$ state in **TPF** computed based on the TD-ωB97XD/6-31G(d) normal modes and energies in the vacuum, solution, and crystal (from left to right).

In this section, we explored the main molecule-centred nonradiative pathways processes in the vacuum and crystal. We first computed the Huang-Rhys factors and the reorganisation energies for S${}_{1}$ to S${}_{0}$ transitions projected on the normal modes (Figure 2).

In **TPF**, similar to other propeller-shaped systems (**TPC** and **TPT**) [9,10], the low-frequency vibrations are hindered by the crystal environment. The modes with ω< 250 cm${}^{-1}$ were considered as low-frequency modes. In the RIM model, it is normally assumed that low-frequency modes are the most important for nonradiative decay. For TPF, these vibrations correspond to collective motions of phenyl-rings with respect to the furan moiety. Their total contributions to the reorganisation energies in the vacuum, solution, and crystal were added showing a significant decrease when going from 952 cm${}^{-1}$ in the vacuum, 1100 cm${}^{-1}$ in THF to 302 cm${}^{-1}$ in the crystal.

According to the RIM model, this effect would lead to less efficient overlap between the vibrational wavefunctions of S${}_{1}$ and S${}_{0}$ and consequently a decrease of intramolecular nonradiative rates and enhancement of the emission quantum yields in the condensed phase. However, the experimental results show that fluorescence is quenched in the solid state. We explored in more detail the intramolecular pathways connecting the optimised critical points in the vacuum and crystal.

**Figure 3:**
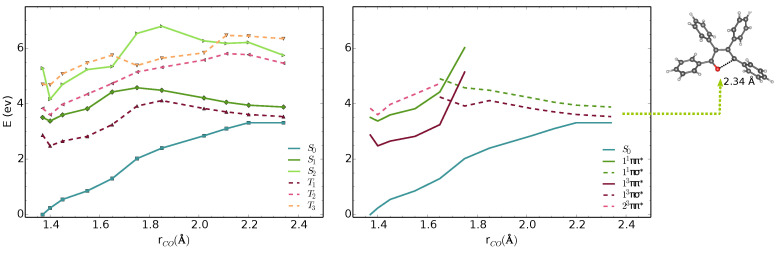
MS-3-CASPT2/CASSCF(10,10)/6-31G(d) energies of S${}_{0}$–S${}_{2}$ and T${}_{1}$–T${}_{3}$ states along interpolated pathway between FC point, S${}_{1}$ minimum and S${}_{1}$–S${}_{0}$ MECI geometry in vacuum. The states are shown in adiabatic (**left**) and diabatic representation (**right**). The diabatic representation was obtained by connecting the excited states corresponding to the same type of transitions along the pathway.

**Figure 4:**
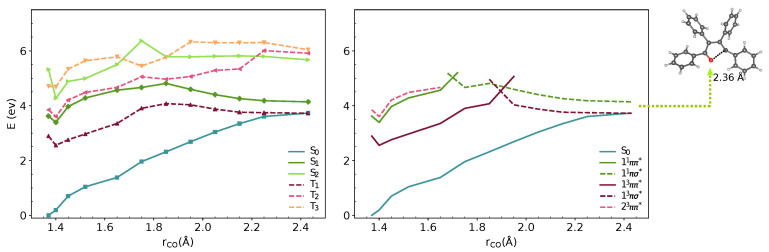
MS-3-CASPT2/CASSCF(10,10)/6-31G(d) energies of S${}_{0}$–S${}_{2}$ and T${}_{1}$–T${}_{3}$ states along interpolated pathway between FC point, S${}_{1}$ minimum and S${}_{1}$–S${}_{0}$ MECI geometry in crystal. The states are shown in adiabatic (**left**) and diabatic representation (**right**). The diabatic representation was obtained by connecting the excited states corresponding to the same type of transitions along the pathway.

We analysed the minimal energy pathways driving the nonradiative decay. In both phases, the optimised S${}_{1}$–S${}_{0}$ MECIs involve ring-opening and C-O bond breaking and occur at C-O distances in the range of 2.3–2.4 Å (Figure 3 and Figure 4). The potential energy profile connecting the FC region with the S${}_{1}$–S${}_{0}$ MECI predicts the crossing of two diabatic states along the C-O stretching coordinate (Figure 3 and Figure 4). There is also a S${}_{1}$–T${}_{1}$ crossing at a similar interatomic distance. In the FC region, S${}_{1}$ has a $\pi \pi^{*}$ character with the electron density localised on the furan moiety and two of the phenyl substituents (Figure 6). This state crosses with a higher-lying $\pi \sigma^{*}$ state at ∼1.7 Å, both in vacuum and crystal. From the initially excited S${}_{1}$, the barrier to the $\pi \pi^{*}$/$\pi \sigma^{*}$ crossing is ≈1.1 eV. Consequently, the S${}_{1}$–S${}_{0}$ MECIs are classically inaccessible in both phases.

Because of the barrier, the system can remain trapped in the $1^{1}\pi \pi^{*}$ minimum. The $2^{3}\pi \pi^{*}$/$1^{1}\pi \pi^{*}$ (T${}_{2}$/S${}_{1}$) intersystem crossing competes with fluorescence from the bright $\pi \pi^{*}$ state. At this geometry, the $1^{1}\pi \pi^{*}$ state is quasi-degenerate with the $2^{3}\pi \pi^{*}$ state, which enhances the probability for the intersystem crossing. We computed the intersystem crossing rates ($k_{ISC}$) for the $2^{3}\pi \pi^{*}$/ $1^{1}\pi \pi^{*}$ (T${}_{2}$/S${}_{1}$) transition based on the Marcus-Levich-Jortner model (Equation (2)) in the vacuum and crystal. Because both states have $\pi \pi {}^{*}$ character, small values of SOCs are expected considering the El-Sayed rule. The values of $\langle S_{1}|H_{SO}|T_{2}\rangle$ obtained with CASSCF(10,10)/6-31G(d) are 0.11 and 0.18 cm${}^{-1}$ in the gas phase and the crystal. The intersystem crossing rates are highly sensitive to small modulations of $\Delta E_{ST}$ that varies significantly with the level of theory. We chose the TD-B3LYP/6-31G(d) value, because its better agreement with experimental emission energies and $\Delta E_{ST}$ (Table 1).

Due to the large differences in their adiabatic energies, the calculated intersystem crossing rates for transition between S${}_{1}$ and T${}_{1}$ states are negligible. The predicted values of $k_{ISC}$ for the transition between T${}_{2}$ and S${}_{1}$ are 1.1 $\times \;10^{8}\;s^{-1}$ and 0.7 $\times \;10^{8}\;s^{-1}$ is the vacumm and solid state respectively (Table 2). After IC from T${}_{2}$, T${}_{1}$ is populated and following vibrational relaxation the system can decay to S${}_{0}$ since the T${}_{1}$–S${}_{0}$ crossing is classically accessible.The analysis of the potential energy surfaces in both phases shows the access to the S${}_{1}$–S${}_{0}$ conical intersection is hindered due to a barrier of more than 1 eV to the $\pi \sigma^{*}$ state. Deactivation through the triplet manifold is facilitated by the ISC in the FC region. However, due to the small values of SOCs, this process is relatively slow. The similar behaviour in both phases does not justify the differences in quantum yields in solution and the solid-state. In the next section, we discuss the effect of crystal environment and specific intermolecular interactions on nonradiative processes in **TPF**.

### 3.3. Crystal Structure: Intermolecular Interactions and Exciton Transport

In comparison to **TPT** [10], the **TPF** molecule features a more planar structure, i.e., at the FC point the side phenyl rings and furan rings form very small dihedral angles in the vacuum, solution and crystal, which is reflected a larger delocalisation of the HOMO and LUMO over these three rings. The phenyl rings attached to C2 and C3 atoms also define small dihedral angles with the furan ring. While in the **TPT** crystal, the close contact between phenyl rings is avoided by significant in-plane slipping of stacked dimers, in the **TPF** crystal the stacked dimers have face-to-tail orientation and in-plane slipping is relatively small in comparison to **TPT**.

**Figure 5:**
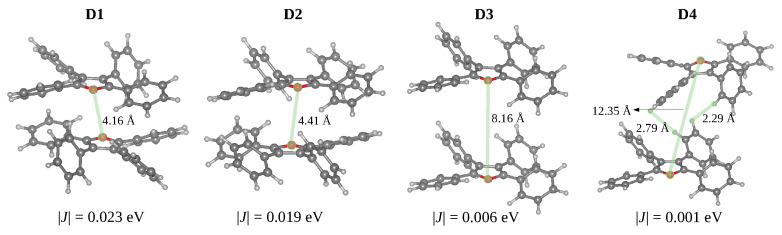
Structures of dimers in the **TPF** showing closest intermolecular contacts (Å) and absolute values of the exciton couplings (in eV).

From the optimised crystal structure, we extracted the dimers with distances between the centroids smaller than 10 Å using fromage. Considering that the oscillator strengths for the $S_{0}$→$S_{1}$ transitions are negligible and for the $S_{0}$→$S_{2}$ transitions are almost twice the values of the excitation in the isolated monomer, all dimers can be classified as H-dimers (Table 3). The stacked dimers (**D1** and **D2**) with a face-to-tail arrangement have the shortest centroid distances. The O-O distances between adjacent layers in the **TPF** crystal (4.15 Å in **D1** and 4.41 Å in **D2** Figure 5) are significantly shorter compared with the S-S distances in **TPT** (6.05 Å), as a result of relatively small in-plane slipping in **TPF**. The **D3** and **D4** dimers feature larger centroid distances and short H..H and C-H..π interactions.

The phenyl substituents do not allow strong π-π stacking interactions and effective exciton couplings. The dimers **D1** and **D2** display stacking between the furan rings with the largest excition couplings of 0.023 and 0.019 eV, respectively. These exciton couplings originate in π-π interactions between transition densities localised on furan and side phenyl rings. For the **D3** and **D4** dimers, the exciton couplings are very small (0.006 and 0.001 eV) due to the large spatial separation of S${}_{1}$ transition densities localised on individual monomers.

**Figure 6:**
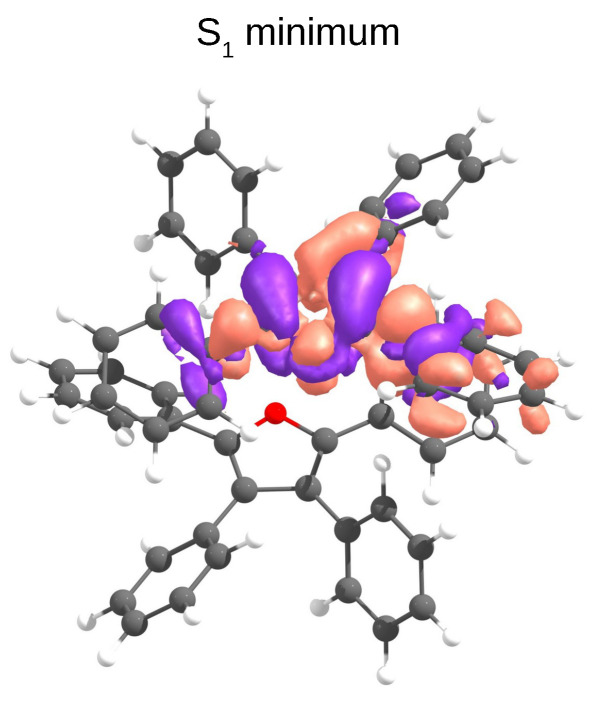
The TD-B3LYP/6-31G(d) density difference of the S${}_{1}$ state at its minimum geometry in the **D1** dimer of **TPF** obtained from QM(TD-B3LYP)/QM’(DFTB) optimisations.

Intermolecular processes, such as exciton hopping, compete with intramolecular relaxation mechanisms in molecular crystals. Shuai et al. have shown that regardless of the nature of the aggregation (J or H), the increase of exciton couplings enhances nonradiative decay rates [7]. We calculated exciton hopping rates ($\nu_{ij}$) between monomers using the Marcus model (Equation (3)). This model is valid in a weak coupling regime, when excitons are localised on individual monomers and transport happens through incoherent hopping, i.e., through exciton hopping events between single molecules. However, in the case when exciton is delocalised over two or more monomer units, this approximation usually predicts overestimated hopping rates [47]. Our calculations show that after relaxation to S${}_{1}$, the electron density localises releasing ≈ 0.4 eV (Figure 6).

According to the Marcus model, the barrier for exciton hopping is approximately λ/4, where λ is the reorganisation energy for S${}_{1}$ to S${}_{0}$ transition [48]. For the **TPF** crystal, the exciton couplings (<0.023 eV) are much smaller in comparison with the reorganisation energy (λ = 0.7 eV), and the exciton transfer is expected to take place in the incoherent regime. The exciton hopping rate in **TPF** between molecules in the dimer **D1**, computed based on Equation (4) is 1.17 $\times \;10^{10}$ $s^{-1}$ and between molecules in the dimer **D2** 7.98 $\times \;10^{9}$ $s^{-1}$. Thermal fluctuations induced by molecular vibrations can modulate the exciton coupling magnitudes and exciton hopping rates.

In comparison with **TPT**, the **TPF** crystal features significantly lower reorganisation energies and slightly larger exciton couplings (Table 4). Both effects result in ∼60 times faster exciton hopping in **TPF** (1.17 $\times \;10^{10}$ in **TPF** vs. 0.02 $\times \;10^{10}$ $s^{-1}$ in **TPT**). In comparison, the exciton hopping is two orders of magnitude slower in the **TPT** crystal (Table 4). We have previously shown that the main nonradiative pathway in **TPT** are localised on monomers and are associated with efficient intersystem crossing channels [10]. Similar nonradiative pathways including internal conversion and intersystem crossing are not energetically accessible in **TPF**, which indicate a significant role of intermolecular exciton mechanisms in the excited state decay of this crystal.

## 4. Conclusions

In contrast to several propeller-shaped systems that show enhanced emission in the solid-state, **TPF** exhibits aggregation quenching. This work highlights the interplay between intramolecular and intermolecular factors in the excited state dynamics of propeller-shaped molecules in the crystalline phase. When the nature of the central atom is modified (Figure 1), moving from C (**TPC**), to S (**TPT**) and O (**TPF**), the main nonradiative pathway changes from puckering to bond breaking [9,10]. Additionally for **TPT** and **TPF**, triplets are essential in the excited-state mechanisms.

In the vacuum and solid-state, the analysis potential energies surfaces of **TPF** shows that the C-O stretching leads to crossings between the excited and ground states. Due to the existence of a barrier of ∼1 eV to reach the $\pi \pi^{*}$/$\pi \sigma^{*}$ crossing, the S${}_{0}$–S${}_{1}$ is inaccessible in both solution and the solid-state. From the S${}_{1}$ minimum, it is possible to populate T${}_{2}$ through ISC. Following IC from T${}_{2}$, T${}_{1}$ is populated and since the S${}_{0}$–T${}_{1}$ crossing is classically accessible, (**TPF**) can decay nonradiatively through the S${}_{0}$–T${}_{1}$ crossing. The slight differences in the potential energy surfaces in the vacuum and solid-state do not justify the significant differences in the experimental quantum yields and the emission quenching in the solid-state.

For both, **TPT** and **TPF**, nonradiative decay pathways involving triplets are accessible in the solid-state, depleting the population of singlets and contributing to a smaller quantum yield in comparison to **TPC**. This is the reason for the weak AIE in **TPT**, however, **TPF** displays quenching in the solid-state. Our calculations indicate that the reason behind the different behaviour of these systems is the activation of intermolecular nonradiative processes in **TPF**.

Because **TPF** has a more planar structure, the crystal packing is more compact enabling more effective interactions between the central aromatic rings. The exciton couplings are slightly larger in **TPF**. Additionally, reorganisation energies are smaller for **TPF** and the exciton hopping rates are much faster in comparison to **TPT**. These transport events will contribute to nonradiative pathways not available in other propeller-shaped systems.

## Figures and Tables

**Table 1:** Vertical absorption and emission energies and oscillator strengths (in parentheses) of the S${}_{1}$ state of **TPF** in the vacuum, solution of THF, and crystal environment.

	Energy (eV)			
	**Vacuum/Solution**		**Crystal**	
	**Absorption**	**Emission**	**Absorption**	**Emission**
RI-CC2/aug-cc-pVDZ	4.10 (0.58)	3.45 (0.71)	-	-
TD-B3LYP/6-31G(d)	3.72 (0.47)	3.16 (0.50)	3.72 (0.41)	3.19 (0.43)
TD-ωB97X-D/6-31G(d)	4.26 (0.51)	3.47 (0.60)	4.26 (0.49)	3.49 (0.52)
TD-ωB97X-D/6-31G(d)/PCM	4.19 (0.63)	3.19 (0.89)	-	-
MS-2-CASPT2/6-31G(d)	3.50	3.14	3.61	3.20
Experimental [11]	3.79	3.24	-	3.24

**Table 2:** Experimental values of fluorescence quantum yield ($\Phi_{f}$), radiative lifetimes ($\tau_{r}$ in ns), fluorescence rates ($k_{r}^{exp}$ in $10^{8}\;s^{-1}$), nonradiative rates ($k_{nr}$ in $10^{8}\;s^{-1}$), and computed fluorescence rates computed based on TD-ωB97X-D/6-31G(d) excitations ($k_{r}^{Ein}$ in $10^{8}\;s^{-1}$) and intersystem crossing rates computed based on TD-B3LYP/6-31G(d) excitations ($k_{ISC}^{MLJ}$ in $10^{8}\;s^{-1}$).

	Solution	Crystal
	Experimental [11]	
$\Phi_{r}$	0.40	0.01
$\tau_{r}$	0.68	0.06
$k_{r}$	5.88	0.67
$k_{nr}$	8.84	1.66 $\times \;10^{2}$
	Predicted	
$k_{r}^{Ein}$	3.93	3.89
$k_{ISC}^{MLJ}$	1.1	0.7

**Table 3:** Excitation energies (*E* in eV), oscillator strengths (*f*) of monomer and relevant dimers and exciton coupling values (*J* in eV) between units in dimers isolated from **TPF** crystal.

Structure	State	*E*	*f*	|J|
Monomer	$S_{1}$ ($\pi \pi^{*}$)	3.9434	0.72	-
D1	$S_{1}$ ($\pi \pi^{*}$)	3.9077	0.00	0.023
	$S_{2}$ ($\pi \pi^{*}$)	3.9544	1.30	
D2	$S_{1}$ ($\pi \pi^{*}$)	3.9209	0.00	0.019
	$S_{2}$ ($\pi \pi^{*}$)	3.9605	1.31	
D3	$S_{1}$ ($\pi \pi^{*}$)	3.9377	0.00	0.006
	$S_{2}$ ($\pi \pi^{*}$)	3.9495	1.40	
D4	$S_{1}$ ($\pi \pi^{*}$)	3.9422	0.00	0.001
	$S_{2}$ ($\pi \pi^{*}$)	3.9437	1.42	

**Table 4:** Reorganisation energies in the crystal ($\lambda^{cr}$ in eV), exciton couplings ($J_{ij}$ in eV) for the dimer with smaller centroid distances, exciton hopping ($\nu_{ij}$ in $10^{10}\;s^{-1}$) in **TPF** and **TPT**. $V_{i}$ are the values of Voronoi volumes of the crystals computed with fromage.

Crystal	$\lambda^{\mathrm{cr}}$	$J_{\mathrm{ij}}$	$V_{i}$	$\nu_{\mathrm{ij}}$
**TPF**	0.70	0.023	1.42	1.17
**TPT**	1.00	0.015	1.35	0.02

## Data Availability

The optimised geometries in the vacuum and the crystal phase can be found in a public repository: https://github.com/Crespo-Otero-group/TPF_data.

## Supplementary Materials

The following supporting information can be downloaded, S1: Geometries, S2: The CASSCF active space orbitals, Figure S1: The CASSCF(10,10)/6-31G(d) orbitals used in the S${}_{0}$/S${}_{1}$ and S${}_{0}$/T${}_{1}$ minimum energy crossing point optimisations and MS-3-CASPT2/SA-3-CASSCF(10,10)/6-31G(d) single point computations along the optimised pathways in the vacuum and crystal.
