# Supplementary material for: Emission Quenching in Tetraphenylfuran Crystal: Why This Propeller-Shaped Molecule Does Not Emit in the Condensed Phase
Source: Molecules. 2022 Jan 14;27(2):522. doi: 10.3390/molecules27020522 (PMC8780143; doi:10.3390/molecules27020522)
Supplement: Supplementary file 1 [file molecules-27-00522-s001.zip › molecules-1527194-supplementary.pdf]

Supporting Information For: Emission quenching in tetraphenylfuran crystal: Why this propeller-shaped molecule does not emit in the condensed phase?

Ljiljana Stojanović, and Rachel Crespo-Otero*

School of Physical and Chemical Sciences,

Queen Mary University of London, Mile End Road, London E1 4NS, UK

E-mail: r.crespo-otero@qmul.ac.uk

Contents

S1 Geometries **3**

S2 The CASSCF active space orbitals **3**

S1 Geometries

The optimised geometries of the ground, excited states, and the minimum energy crossing points between the ground state and S_1 state and the ground state and T_1 state discussed in the main paper are deposited at: https://github.com/Crespo-Otero-group/TPF_data

S2 The CASSCF active space orbitals

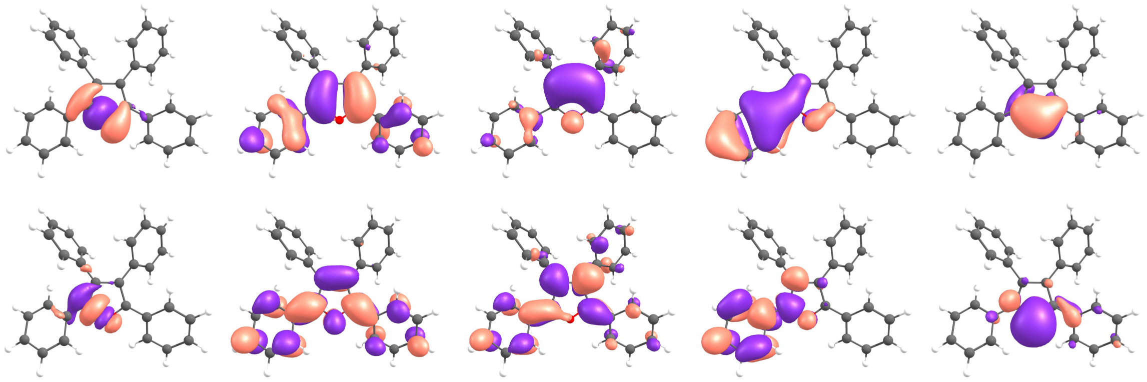

Figure S1: The CASSCF(10,10)/6-31G(d) orbitals used in the S_0/S_1 and S_0/T_1 minimum energy crossing point optimisations and MS-3-CASPT2/SA-3-CASSCF(10,10)/6-31G(d) single point computations along the optimised pathways in the vacuum and crystal.
